# Attitudes to Mental Illness and Its Demographic Correlates among General Population in Singapore

**DOI:** 10.1371/journal.pone.0167297

**Published:** 2016-11-28

**Authors:** Qi Yuan, Edimansyah Abdin, Louisa Picco, Janhavi Ajit Vaingankar, Shazana Shahwan, Anitha Jeyagurunathan, Vathsala Sagayadevan, Saleha Shafie, Jenny Tay, Siow Ann Chong, Mythily Subramaniam

**Affiliations:** Research Division, Institute of Mental Health, Singapore; Universidade Federal do Rio de Janeiro, BRAZIL

## Abstract

**Background:**

Public attitudes to mental illness could influence how the public interact with, provide opportunities for, and help people with mental illness.

**Aims:**

This study aims to explore the underlying factors of the Attitudes to Mental Illness questionnaire among the general population in Singapore and the socio-demographic correlates of each factor.

**Methods:**

From March 2014 to April 2015, a nation-wide cross-sectional survey on mental health literacy with 3,006 participants was conducted in Singapore.

**Results:**

Factor analysis revealed a 4-factor structure for the Attitudes to Mental Illness questionnaire among the Singapore general population, namely social distancing, tolerance/support for community care, social restrictiveness, and prejudice and misconception. Older age, male gender, lower education and socio-economic status were associated with more negative attitudes towards the mentally ill. Chinese showed more negative attitudes than Indians and Malays (except for prejudice and misconception).

**Conclusions:**

There is a need for culture-specific interventions, and the associated factors identified in this study should be considered for future attitude campaigns.

## Background

Attitudes towards people with mental disorders refer to individual beliefs about what people with mental illness are like and how they should be treated [1, 2]. Previous studies have shown that these attitudes could vary from acceptance [3], tolerance [4], to stigma [5], and even fear [6]. Researchers have proposed two different ways by which public attitudes might affect individuals with mental illness. The first relates to how the public might interact with, provide opportunities for, and help support a person with mental illness [7]. In this case, the public refers to both the general public such as the community and institutions like hospitals, as well as co-workers, friends and family members of people with mental illness [8]. When attitudes and beliefs are expressed positively, they can lead to supportive and inclusive behaviours (e.g. willingness to hire a person with mental illness); but when they are expressed negatively, they can cause avoidance, exclusion from daily activities, exploitation, and discrimination [7]. The second frames how people with mental illness experience and express their own psychological problems and whether they are willing to disclose their symptoms and seek help [7, 8]. Rüsch et al. [9] reported that after controlling for demographics, people’s intentions to seek help were positively associated with the tolerance and support for community care of mental illness.

During the past few decades, there has been an increase in mental health literacy among the general public, especially regarding knowledge of the biological and genetic basis of mental illness [3, 10]. However, such increases have not lessened the stigmatization and discrimination towards people with mental illness [10]. As a cluster of negative attitudes and beliefs, stigma is a concept which has received a lot of research attention [5, 7, 11]. Although there are different tools that can measure stigma [12, 13], public attitude is a much broader issue than these negative attitudes. Other attitudes like tolerance [4] and acceptance [3] are also part of it. Thus there is a need for multidimensional attitudes scales for assessing public attitudes to mental illness. The Attitudes to Mental Illness (AMI) questionnaire is a tool developed by the UK Department of Health, adapted from the 40-item Community Attitudes toward the Mentally Ill Scale [14]. As a multidimensional instrument, it covers four aspects of public attitudes, namely fear and exclusion of people with mental illness, understanding and tolerance of mental illness, integrating people with mental illness into the community, and causes of mental illness and the need for special service [15]. The UK Department of Health has used it to assess public attitudes among the UK general population for more than 20 years [16], and it showed good internal reliability (Cronbach’s alpha = 0.87 [17]). Another 2-factor structure was also identified, which included ‘prejudice and exclusion’ (Cronbach’s alpha = 0.83), and ‘tolerance and support for community care’ (Cronbach’s alpha = 0.77) [9]. Given that these were based on the UK general population, it is therefore unknown whether these two different factor structures are applicable to the multi-ethnic population in Singapore.

Previous studies suggested that socio-demographic characteristics could affect public attitudes. Lauber et al. [18] found that demographic factors such as age, gender and the cultural background contributed to social distances, with the explained variance as 44.8%. Another study among Mexican Americans found that demographics such as age, gender and education were the primary variables associated with attitudes towards mental illness [4]. Moreover, the direction of such relationships may vary across different cultures—a Swiss sample suggested that women tended to have more negative attitudes towards people who are mentally ill [19]. While it was reversed among a sample from Germany, where female respondents were more accepting of people with mental illness [20]. Locally, a study on general attitudes to mental illness [21] found various socio-demographic correlates relating to attitudes including age and education; however, given this study was conducted over 10 year ago, there is a need for more up-to-date evidence. A more recent national study was conducted, which explored stigma (personal stigma and social distancing) and its correlates among the Singapore general population, specifically in relation to five mental disorders [13]. This study also found that age, gender, ethnicity, education and income level were correlated with personal stigma towards people with mental illness.

The current study aimed: 1) to explore the factor structure of the AMI questionnaire among the multi-ethnic general population in Singapore; and 2) to explore the socio-demographic correlates of each AMI factor and identify how these characteristics affect public attitudes to mental illness among this population.

## Methods

### Procedures

Data for the current study were from a nation-wide cross-sectional survey of mental health literacy conducted in Singapore from March 2014 to April 2015. It adopted a disproportionate stratified sampling design with 12 strata by age (18–34, 35–49, 50–65) and ethnicity groups (Chinese, Malay, Indian, and other ethnic groups). A probability sample was randomly selected via a registry that maintains the names and socio-demographic characteristics such as age, gender, ethnicity and household address of all residents in Singapore. To be included in this study, participants had to be Singapore residents (Citizens or Permanent Residents) aged between 18–65 years and living in Singapore during the recruitment period. Residents aged 50–65 years, Malays and Indians were over-sampled to ensure sufficient sample size for subgroup analysis. More detailed information on the sampling strategy is found in the paper by Chong et al. [22].

Face-to-face interviews were conducted by trained interviewers; and the respondent could choose the language for the interview: English, Chinese, Malay or Tamil. Data was captured via iPad, which was programmed to display a dual language screen, allowing bi-lingual interviewers to translate terms or phrases as required (by the respondent) in a consistent way; this method minimized the potential for misinterpretation or ad hoc translations by interviewers. Individuals who were out of the country during the recruitment period, unable to be contacted due to incomplete or incorrect addresses, and unable to complete the interview in one of the specified languages were excluded from the current study. In total 4,231 people were contacted, of which 3,006 completed the survey which yielded an overall response rate of 71.1%.

The study was approved by the National Healthcare Group Domain Specific Review Board in Singapore. Written informed consent was obtained from all participants aged 21 years and above as well as from parents or guardians of participants who were 18–20 years old.

### Measurements

Twenty-six items of the original 27-item AMI questionnaire were used in the current study to measure public attitudes towards mental illness. The single item ‘most women who were once patients in a mental hospital can be trusted as babysitters’ was excluded due to its low loading (below 0.3) in a previous study [9]. Items were rated on a 5-point Likert scale ranging from ‘1 = strongly agree’ to ‘5 = strongly disagree’. The original scale was proposed to cover 4 components: fear and exclusion of people with mental illness, understanding and tolerance of mental illness, integrating people with mental illness into the community, and causes of mental illness and the need for special service [15].

Socio-demographic information including age, gender, ethnicity, marital status, education level, employment status and personal monthly income were also collected.

### Translation and Cognitive Testing

The survey measures were translated into Mandarin, Malay and Tamil. To ensure the conceptual equivalence of the instruments in the different languages, the whole translation procedure followed a process that was adapted from guidelines of the World Health Organization, which had been used among two previous national studies in Singapore [22, 23]. This included 1) single forward translation by a professional firm; 2) review by an expert panel comprising the professional translators, content experts and a layperson to identify and resolve any inadequate expressions in the translation and discrepancies between the translated and original version; 3) pre-testing and cognitive interviews among individuals representing the target population in term of the age-groups, gender, ethnicities, and socio-demographics; and 4) development of the final version [22].

The cognitive interviews refer to a common means of applying the cognitive model in a manner that may ultimately improve the quality of survey questions through the study of comprehension, retrieval, judgment, and response processes [24]. During this process, respondents were interviewed by trained researchers who systematically probed on whether they could repeat the questions and what came to their mind when they heard a particular phrase or term and they were asked how they decided on their response. Respondents also reported any word they did not understand and any word or expression that they found offensive or unacceptable; and where alternative words or expressions exist for one item or expression, the respondent was asked which of the alternatives conforms better to their usual language. Minor changes were made to the questions based on the cognitive interview findings; this was to ensure the items of the instrument would be understood in the manner they were intended to be and to avoid potential misinterpretation. More information on this process is available in a previous publication [25].

### Data Analyses

All estimates were weighted to adjust for over sampling and post-stratified for age and ethnicity distributions between the survey sample and the Singapore resident population in 2012. Descriptive analysis was conducted for the socio-demographic variables. Weighted mean and standard error (SE) were presented for continuous variables; while for categorical variables, they were presented as frequencies and percentages.

The factor structure was determined by several steps. First of all, using confirmatory factor analysis (CFA), we tested the 4-factor and 2-factor structures of AMI from previous studies [9, 15]. However, the model fit indices suggested that these two models didn’t fit into our data. Therefore, following previous studies [13, 25], we randomly split the weighted dataset into 2 separate parts with equal number of observations for each (n = 1,503). Exploratory factor analysis (EFA) was conducted for the first half-dataset. Eigenvalues > 1.0, scree plot, pattern of loadings on each item (e.g. cross-loading), and the interpretability were used to determine the appropriate number of factors that should be extracted. To allow the correlation between factors, oblique promax rotation was used, and the factor loading cut-off was set as 0.4. This was followed by a CFA for the second half-dataset to confirm this factor structure derived from EFA. A good model was defined as 1) the comparative fit index (CFI) > 0.95; 2), the Tucker-Lewis index (TLI) >0.95, and 3) the root mean square error of approximation (RMSEA) <0.06 [26]. All structural equation modelling analyses were performed on polychoric correlation matrix using Mplus version 7.0 with the weighted least squares with mean and variance adjusted estimator for categorical variables [27]. The internal reliability (Cronbach’s alpha) for each factor was calculated as well.

Multivariate linear regression was conducted to examine the socio-demographic correlates (i.e. age, gender, ethnicity, marital status, education level, employment status and personal income) for each of the AMI factor scores (dependent variables). A two-sided p-value below 0.05 was considered as statistically significant. The descriptive and the multivariate linear regression analyses were conducted using SAS 9.3.

## Results

The results of the descriptive analysis are listed in Table 1. The participants had an average age of 40.9 (SE = 0.11), with almost equal percentage of both genders (male = 50.9%). Chinese made up about three quarters of the participants (74.7%), followed by Malays (12.8%) and Indians (9.1%). About 64% of the participants were married, compared to 31.4% who were never married. The education level of the participants tended to be moderately high; with 31.3% having a diploma and 29.6% having a university degree. Most of the participants were employed (77.6%), followed by housewives/homemakers (8.7%) and students (6.7%). The unemployment rate of the current sample was quite low, at around 4%. The majority of respondents had a monthly salary lower than SGD 6,000 (86.9%).

**Table 1:** Socio-demographic characteristics of the study participants.

	N	Weighted %	SE
**Age Group**			
18–34 years	1152	34.4	0.04
35–49 years	896	35.2	0.04
50–65 years	958	30.5	0.06
**Gender**			
Female	1506	49.1	1.25
Male	1500	50.9	1.25
**Ethnicity**			
Chinese	1034	74.7	0.04
Malay	977	12.8	0.01
Indian	963	9.1	0.01
Others	32	3.3	0.04
**Marital Status**			
Married	1916	64.0	1.01
Never married	927	31.4	0.93
Others (divorced, widowed, separated)	162	4.6	0.51
**Education Level**			
Primary and below	431	13.4	0.78
Secondary education include O/N level	820	25.8	1.04
A level, polytechnic and other diploma	999	31.3	1.12
University	756	29.6	1.12
**Employment Status**			
Employed	2227	77.6	0.96
Housewife/homemaker	378	8.7	0.62
Retired	78	3.0	0.42
Student	203	6.7	0.53
Unemployed	120	4.0	0.48
**Personal Income**			
< SGD 2,000	1346	40.5	1.18
SGD2,000-SGD5,999	1162	46.4	1.26
SGD6,000 or above	294	13.1	0.91

To explore the factor structure of the AMI, CFA was performed to confirm whether the 4-factor and 2-factor structures identified from previous studies [9, 15], were best suited to the current sample. However, results suggested that both of them had poor fit—for the 4-factor model [15], χ^2^_(df)_ = 1325.167 _(128)_, CFI = 0.802, TLI = 0.878, and RMSEA = 0.056; for the 2-factor model [9], χ^2^_(df)_ = 1833.422 _(129)_, CFI = 0.718, TLI = 0.828, and RMSEA = 0.066. In this case, EFA analysis was conducted for the first half-dataset (n = 1,503). The eigenvalues and scree plot on all 26 items of the questionnaire suggested that 4-, 5-, or 6-factor models were all potential solutions. During the analysis, factor loadings of each item in all 3 models were explored. Items were excluded based on the following priority: items have 1) consistently lowest loading across all models; 2) consistent cross-loading across all models; 3) lowest loading across different models; 4) lowest loading; and 5) cross-loading. After removing 2 items, the eigenvalues and scree plot suggested that the 6-factor model was no longer suitable; thus for the following steps, only the 4- and 5-factor models were considered. After removing another 2 items, the 5-factor model became unstable (one item had a factor loading above 1). In the end, a 4-factor structure comprising 20 items was selected, and it had a good model fit (χ^2^_(df)_ = 213.808 _(116)_, RMSEA = 0.024). The removed items are shown in S1 Appendix.

The four extracted factors were subsequently reviewed and labelled—factor 1 (3 items) was named ‘social distancing’; factor 2 (9 items) was named ‘tolerance/support for community care’, since it shared almost all items with the same factor identified by Rüsch et al. [9] (except the item ‘increased spending on mental health services is a waste of money’); factor 3 (3 items) as ‘social restrictiveness’; and factor 4 (5 items) as ‘prejudice and misconception’. Refer to Table 2 for more information on the 4-factor model. CFA was conducted to test the model fit of this 4-factor model among the second half-dataset (n = 1,503), and the result suggested that the model was acceptable (χ^2^_(df)_ = 236.727 _(80)_, CFI = 0.933, TLI = 0.955, and RMSEA = 0.036). The internal reliability statistics for the four factors were 0.707, 0.696, 0.709, and 0.665, respectively.

**Table 2:** Factor loadings from EFA analysis on Attitudes to Mental Illness questionnaire (n = 1,503).

Item Description	Factor Loading			
	f1	f2	f3	f4
**Factor 1- Social Distancing**				
AMI-1 Having mental health facilities in a residential area downgrades the neighbourhood	0.450			
AMI-2 It is frightening to think of people with mental problems living in our neighbourhoods	0.965			
AMI-3 I would not want to live next door to someone who has been mentally ill	0.665			
**Factor 2 –Tolerance/Support for community care**				
AMI-9 We have a responsibility to provide the best possible care for people with mental illness		-0.588		
AMI-10 Anyone can become mentally ill		-0.562		
AMI-11 Increased spending on mental health services is a waste of money		0.580		
AMI-13 We need to adopt a more tolerant attitude toward people with mental illness in our society		-0.673		
AMI-15 As far as possible, mental health services should be provided through community based facilities such as policlinics, GPs and family counselling services.'		-0.567		
AMI-16 'People with mental illness are not as dangerous as most people think they are'		-0.437		
AMI-18 The best therapy for many people with mental illness is to be part of a community		-0.652		
AMI-19 Residents should not be afraid of visiting mental health services in their neighbourhood		-0.676		
AMI-22 No-one has the right to exclude people with mental illness from their neighbourhood		-0.601		
**Factor 3 –Social Restrictiveness**				
AMI-5 Anyone with a history of mental problems should be excluded from the public/civil service			0.630	
AMI-6 People with mental illness should not be given any responsibility			0.782	
AMI-7 People with mental illness are a burden on society			0.638	
**Factor 4—Prejudice and Misconception**				
AMI-8 As soon as a person shows signs of mental disturbance, they should be hospitalized				0.424
AMI-23 Mental hospitals are the only means of treating people with mental illnesses				0.533
AMI-24 There are sufficient existing services for people with mental illness				0.717
AMI-25 One of the main causes of mental illness is a lack of self-discipline and will-power				0.517
AMI-26 There is something about people with mental illness that makes it easy to identify them from normal people				0.492

To enable easier interpretation of the regression results, the selected items of AMI were reverse scored (changed to ‘1 = strongly disagree’ to ‘5 = strongly agree’), following Rüsch et al. [9]. The total score of each factor was then summed up and used in the multivariate regression analyses. For factor 2, the scoring of the single item with positive factor loading was reverse scored again, and then added up with the scores of the remaining items within this factor. In this case, more positive attitudes towards people with mental illness were characterized as—lower ‘social distancing’, higher ‘tolerance/support for community care’, lower ‘social restrictiveness’, and lower ‘prejudice and misconception’ scores. The average factor score for ‘social distancing’ was 8.07(SE = 0.07, range 3 to15), 14.81 for ‘tolerance/support for community care’ (SE = 0.10, range 9 to 45), 7.21 for ‘social restrictiveness’ (SE = 0.07, range 3 to 15), and 15.36 for ‘prejudice and misconception’ (SE = 0.10, range 5 to 25).

Multivariate linear regression analyses results suggested that age, gender, ethnicity, marital status, education level, employment status and personal income were all significantly associated with the AMI factors (Table 3). Those aged between 35–65 years, with relatively lower education (i.e. secondary education including O/N level, or below), and being a housewife/homemaker were significantly associated with higher ‘social distancing’; while any ethnicity other than Chinese was significantly associated with lower ‘social distancing’. Female gender, Indian ethnicity, and being unemployed were associated with more ‘tolerance/support for community care’; while lower education, and being a housewife/homemaker were negatively associated. ‘Social restrictiveness’ was positively associated with those aged between 35–65 years, education level lower than university, and being a housewife/homemaker; while negatively associated with being a female, Malay or Indian ethnicity, and being a student. Lastly, ‘prejudice and misconception’, was positively associated with those aged 50–65 years, Malay or Indian ethnicity, education level lower than university, and a monthly income less than SGD 2,000; on the other hand, it was negatively associated with female gender, never married, and being a student or unemployed.

**Table 3:** Socio-demographic correlates of Attitudes to Mental Illness questionnaire.

	Social distancing				Tolerance/Support for community care				Social restrictiveness				Prejudice and Misconception			
	β	95% CI		*p*	β	95% CI		*p*	β	95% CI		*p*	β	95% CI		*p*
**Age Group**																
50–65 years	1.000	0.534	1.467	**<.0001**	0.057	-0.571	0.684	0.859	1.838	1.397	2.279	**<.0001**	1.183	0.572	1.794	**0.0002**^a^
35–49 years	0.595	0.198	0.992	**0.003**	0.189	-0.321	0.698	0.468	1.091	0.725	1.457	**<.0001**	0.239	-0.301	0.778	0.386
18–34 years	Ref				Ref				Ref				Ref			
**Gender**																
Female	0.052	-0.249	0.353	0.735	0.519	0.112	0.926	**0.013**	-0.482	-0.759	-0.206	**0.001**	-0.382	-0.764	-0.001	**0.0495**^b^
Male	Ref				Ref				Ref				Ref			
**Ethnicity**																
Others	-2.190	-3.095	-1.285	**<.0001**	0.698	-0.443	1.839	0.230	-1.234	-2.597	0.130	0.076	0.217	-1.175	1.610	0.759
Indian	-0.433	-0.723	-0.144	**0.003**	0.414	0.051	0.777	**0.026**	-0.296	-0.555	-0.037	**0.025**	1.286	0.926	1.645	**<.0001**
Malay	-0.298	-0.576	-0.020	**0.036**	-0.208	-0.589	0.174	0.286	-0.297	-0.556	-0.038	**0.025**	1.034	0.691	1.378	**<.0001**
Chinese	Ref				Ref				Ref				Ref			
**Marital Status**																
Others (divorced, widowed, separated)	0.333	-0.358	1.025	0.345	0.083	-0.858	1.023	0.863	0.436	-0.270	1.143	0.226	0.494	-0.436	1.424	0.298
Never married	-0.228	-0.613	0.156	0.245	-0.162	-0.688	0.363	0.545	-0.261	-0.613	0.090	0.145	-0.524	-1.036	-0.011	**0.045**
Married	Ref				Ref				Ref				Ref			
**Education Level**																
Primary and below	0.639	0.017	1.261	**0.044**	-2.192	-3.038	-1.345	**<.0001**	1.957	1.372	2.543	**<.0001**	4.271	3.501	5.042	**<.0001**
Secondary education include O/N level	0.514	0.023	1.006	**0.040**	-1.306	-1.936	-0.677	**<.0001**	1.006	0.547	1.465	**<.0001**	3.249	2.642	3.855	**<.0001**
A level, polytechnic and other diploma	0.261	-0.131	0.654	0.192	-0.458	-0.968	0.051	0.078	0.609	0.229	0.989	**0.002**	1.667	1.150	2.184	**<.0001**
University	Ref				Ref				Ref				Ref			
**Employment Status**																
Unemployed	0.136	-0.761	1.033	0.766	1.312	0.193	2.431	**0.022**	-0.370	-1.242	0.502	0.406	-1.246	-2.201	-0.291	**0.011**
Student	-0.586	-1.196	0.024	0.060	0.710	-0.097	1.518	0.085	-0.665	-1.170	-0.161	**0.010**	-1.769	-2.522	-1.017	**<.0001**
Retired	-0.098	-1.260	1.063	0.868	0.202	-0.875	1.280	0.713	-0.188	-1.200	0.823	0.715	-0.820	-1.880	0.240	0.130
Housewife/homemaker	0.929	0.332	1.527	**0.002**	-1.009	-1.861	-0.157	**0.020**	0.587	0.010	1.165	**0.046**	0.334	-0.497	1.165	0.431
Employed	Ref				Ref				Ref				Ref			
**Personal Income**																
< SGD 2,000	-0.598	-1.214	0.017	0.057	-0.477	-1.254	0.300	0.228	0.257	-0.295	0.808	0.361	0.935	0.147	1.724	**0.020**
SGD2,000-SGD5,999	-0.091	-0.625	0.443	0.738	0.022	-0.628	0.673	0.946	0.326	-0.137	0.789	0.167	0.473	-0.180	1.126	0.156
SGD6,000 or above	Ref				Ref				Ref				Ref			

^a^ p = 0.0002;

^b^ p = 0.0495; Ref—reference group

More positive attitudes towards people with mental illness were characterized as—lower ‘social distancing’, higher ‘tolerance/support for community care’, lower ‘social restrictiveness’, and lower ‘prejudice and misconception’.

## Discussion

Unlike the two different factor structures of AMI from previous studies [9, 15], our results suggested a 4-factor structure with 20 items among the Singapore general population. The first factor is about ‘social distancing’–people’s intention to distance themselves from individuals with mental illness in their community. The second factor relates to understanding and tolerance towards the mentally ill and the intention of integrating mental health service into the community (i.e. ‘tolerance/support for community care’). The third factor is about limiting the societal roles and responsibilities of those with mental illness (i.e. ‘social restrictiveness’); while the last factor relates to public’s ‘prejudice and misconception’ over mental illness. CFA confirmed that this model had an acceptable fit, with only the CFI being slightly lower than the recommended cut-off of 0.95 [26]. In this sense, the multidimensionality of public attitudes to mental illness was confirmed.

Further analyses on the socio-demographic correlates of the four AMI factors suggested that the relationships between several socio-demographic characteristics and attitudes were quite consistent across different factors. Firstly, people belonging to the older age group (50–65 years) generally had more negative attitudes towards the mentally ill, and this applied for ‘social distancing’, ‘social restrictiveness’ and ‘prejudice and misconception’. A literature review on public attitudes towards mental illness suggested that out of the 33 studies, 32 reported positive associations between negative attitudes and age [28]. Similar findings were also reported in local studies, with younger adults being more tolerant [21] and less stigmatising [13]. This might be due to improved public knowledge of mental illness [10], and the development of information technology which makes such knowledge easily accessible especially for young adults who are more familiar with the use of such technology. Gender was another factor affecting public attitudes. The relationship between gender and public attitudes could vary due to different reasons such as cultural differences [19, 20]. A previous local study on stigma suggested that female gender was likely to be associated with lower stigma among the general population in Singapore [13]. A 2006 review also summarized that females reported more positive attitudes to mental illness among more than half of the studies which had explored this issue [28]. This is similar to our findings, where females showed more tolerance and support for community care, less social restrictiveness, and less prejudice and misconception towards people with mental illness. Although some studies also indicated that females tended to desire less social distance than males [13, 29, 30]; this relationship was not evident among our sample. Potential reasons could be that items used to measure social distance in our instrument were about the distance in the neighbourhood or community; however, for other studies, they focused on social distance in terms of personal contact with individuals with mental illness [13, 30]. Lower education was also found to be consistently associated with more negative attitudes to mental illness across all four factors of AMI. This finding has been reported by other studies as well [13, 28, 31], and it suggests that individuals with higher education had greater knowledge relating to mental illness. Another explanation could be that people with higher education have more access to health information, or they have a better understanding of such information as a result of their higher education.

An interesting finding lies in the correlation between ethnicity and AMI factors. In our study, as compared to Chinese, both Malays and Indians had higher prejudice and misconception towards mental illness; but they also had less social distancing and less social restrictiveness towards those who were mentally ill. Meanwhile, Indians tended to have higher tolerance and support for community care of people with mental illness. Prejudice and misconception is a factor highly correlated with people’s knowledge of mental illness. In this case, possessing less knowledge of mental illness didn’t necessarily mean that individuals would have more social distancing, less tolerance/support to community care, and more social restrictiveness towards people with mental illness; other factors such as cultural differences might also play an important role in this process. This could be particularly true for Malays. In Islam, the religion practiced by the large majority of Malays in Singapore, mental illness is perceived as a test from God [32, 33], and illness could be treated as an opportunity to remedy disconnection from God or resolve a lack of faith through regular prayer and a sense of self-responsibility [34, 35]. Thus they were more tolerant to mental illness, as reported in other local studies as well [13, 21]. Alternatively, such differences might also be caused by people’s misunderstanding on the scales items. In another study among the same study sample, by providing detailed vignettes on different mental health problems, Malays and Indians tended to view mental illness as weakness but not illness [13]. In our measurement, the wording ‘mental illness’ might have misled the participants while they were answering the questionnaire. Although Malays and Indians scored higher on ‘prejudice and misconception’ in our study, they still tended to view mental illness as weakness subconsciously and thus showed more tolerance to those people. A previous study among rural Indian citizens suggested that Indians were generally willing to be a friend, neighbour or workmate of the mentally ill; and the belief that ‘agreeing mental problems were caused by personal weakness’ was positively associated with their intention to reduce social distance [36]. These two explanations are a bit contradictory and hence, further studies are needed to test and clarify the differences, and to explore the underlying mechanisms. However, both explanations suggest that compared to Chinese, Malays and Indians in Singapore lack knowledge of mental illness, which indicates a need for future informational campaigns.

Other predictors of public attitudes included marital status, employment status, and personal income. Being a housewife/homemaker was associated with more social distancing, less tolerance and support for community care, and higher social restrictiveness towards mentally ill. Being unemployed was associated with higher tolerance and support for community care; and being a student predicted less social restrictiveness towards people with mental illness. Being unmarried, unemployed, and being a student were associated with less prejudice and misconception towards those with mental illness; while having a monthly income less than SGD 2,000 predicted more prejudice and misconception. Marital status is usually related to age, with younger adults being less likely to be married [25]; this also applies to individuals who were students. In this sense, the association between being unmarried or a student and ‘prejudice and misconception’ might be simply caused by the effect of age. Further examination of our data suggested that about 88% of the housewives/homemakers in our study had a monthly income lower than SGD 2,000. In this case, being a housewife/homemaker or having a monthly income less than SGD 2,000 could be viewed as lower socio-economic status. This is consistent with findings that suggest people with low socio-economic status are much less tolerant of mentally ill patients [37, 38]. Our study also found that being unemployed was correlated with more positive attitudes towards mental illness. However, this is not consistent with findings from another study which found adults who were unemployed or unable to work to be more likely to show negative attitudes [7].

There are some limitations to the study. Firstly, the cross-sectional design precluded us from drawing conclusions on causal-relationships. Secondly, although the interviewer-administered questionnaire could ensure the response quality, respondents might alter their response to avoid embarrassment in the presence of an interviewer [39] or they may be reluctant to reveal beliefs unlikely to be endorsed by the interviewer i.e. there may have been some social desirability bias [40]. Thirdly, previous studies suggested that people tended to have different attitudes towards different mental illnesses [28]. However, in our study, the participants were asked to answer the questionnaire based on the general term, ‘mental illness’. As a result, their responses were highly dependent on how they interpreted this term, and thus might be inconsistent across the sample. Lastly, although several robust strategies were employed to ensure the conceptual equivalency of the assessment instruments in different languages, it is still possible that languages differences contributed to some of the significant findings in our study (e.g. difference by ethnicity groups).

These limitations notwithstanding, this is the first study that has systematically studied AMI outside UK. Based on rigorous methodologies, a different factor structure was identified among the Singapore sample, which indicates potential cultural differences between Western and Asian populations in their perceptions of mental illness. It also has a large sample size with a good overall response rate (71.1%) which is representative of the general population. Lastly, before applying the questionnaire to the local population, necessary changes were made based on cognitive interviews to ensure its comprehension and local relevance.

## Conclusion

The current study confirmed the multidimensionality of the public’s attitudes to mental illness, and it also identified several key characteristics associated with negative attitudes to mental illness. Risk factors for negative attitudes include older age, male gender, Chinese ethnicity, lower education and lower socioeconomic status. For Indian and Malay ethnicities, although they tended to show more positive attitudes on social distancing, tolerance/support for community care, and social restrictiveness; they also had more prejudice and misconception towards the mentally ill. Tolerance or support to community care was positively associated with being female, Indian and with higher education level; while negatively associated with being a housewife or homemaker. The potential difference between Western and Asian population in how they perceived mental illness also suggested that there is need for well-planned and culturally sensitive public attitude campaigns. Future studies could explore cultural differences and how these differences might affect public attitudes.

## Supporting Information

S1 Appendix List of AMI items excluded. (PDF)
