# Supplementary material for: Attitudes to Mental Illness and Its Demographic Correlates among General Population in Singapore
Source: PLoS One. 2016 Nov 28;11(11):e0167297. doi: 10.1371/journal.pone.0167297 (PMC5125689; doi:10.1371/journal.pone.0167297)
Supplement: S1 Appendix — (PDF) [file pone.0167297.s001.pdf]

S1 Appendix. List of AMI items excluded.

(Following the exclusion order in the analysis)

AMI- 14 People with mental illness are often made fun of.

AMI – 17 More should be done to protect the public from people with mental illness.

AMI – 21 Mental illness is an illness like any other.

AMI – 4 It would be foolish to marry a person who has suffered from mental illness, even though they seem fully recovered.

AMI – 20 People with mental health problems should have the same rights to a job as anyone else.

AMI – 12 People with mental illness don't deserve our understanding.
